# Generation and Storage of Random Voltage Values via Ring Oscillators Comprising Feedback Field-Effect Transistors

**DOI:** 10.3390/nano14070562

**Published:** 2024-03-23

**Authors:** Jaemin Son, Juhee Jeon, Kyoungah Cho, Sangsig Kim

**Affiliations:** Department of Electrical Engineering, Korea University, 145 Anam-ro, Seongbuk-gu, Seoul 02841, Republic of Korea; jaeminson@korea.ac.kr (J.S.); isdf35@korea.ac.kr (J.J.)

**Keywords:** field-effect transistor, positive feedback loop, oscillator, physical unclonable function, random number generator

## Abstract

In this study, we demonstrate the generation and storage of random voltage values using a ring oscillator consisting of feedback field-effect transistors (FBFETs). This innovative approach utilizes the logic-in-memory function of FBFETs to extract continuous output voltages from oscillatory cycles. The ring oscillator exhibited uniform probability distributions of 51.6% for logic 0 and 48.4% for logic 1. The generation of analog voltages provides binary random variables that are stored for over 5000 s. This demonstrates the potential of the ring oscillator in advanced physical functions and true random number generator technologies.

## 1. Introduction

Modern computing systems have witnessed rapid development with data-intensive applications, such as artificial intelligence and the Internet of Things [1,2]. However, significant security problems arise, as vast amounts of data are digitally stored in the memory units of computers, where the data are easily accessible and thereby vulnerable to cyberthreats [3]. A practical method to protect data from cyberattacks is to utilize physical unclonable functions (PUFs) or true random number generators (TRNGs), which provide cryptographic keys [4,5]. Unlike data security algorithms, PUFs and TRNGs produce unpredictable cryptographic values because of the inherent randomness of their component devices. This randomness primarily originates from fabrication process variations and stochastic mechanisms that cannot be physically duplicated or cloned [6]. Therefore, PUFs and TRNGs are the key building blocks in the design of security systems.

Recently, nonvolatile random-access memories (RAMs), including ferroelectric RAMs (FRAMs) [7,8], magnetic RAMs (MRAMs) [9,10,11], and resistive RAMs (ReRAMs) [12,13,14], have been introduced to generate random variables by exploiting inherent stochastic phenomena. Nonvolatile RAMs utilize various stochastic parameters, such as random telegraph noise, internal latency variations, cycle-to-cycle variations, and device-to-device variations. The unpredictability of the variables and operational stability should be ensured when using their stochastic parameters as sources of random variables. The stochastic nature of nonvolatile RAMs improves the entropy rates of random variables compared with PUFs and TRNGs. However, this deteriorates the device performance and stability during cycling [15]. In particular, FRAMs and ReRAMs suffer from low cycle-to-cycle endurance properties, which leads to a loss of probability distribution for random variables. The thermal instabilities and high energy consumption of MRAMs because of their high current density remain challenging, although they have better cycle-to-cycle endurance than other nonvolatile RAMs.

Feedback field-effect transistors (FBFETs) are emerging as a solution for PUF and TRNG applications because of their exceptional stability under both operational and environmental conditions [16,17,18]. They operate with logic-in-memory (LIM) functions because of their unique positive feedback mechanism, which allows their logic circuits to store logical states [19,20,21]. In particular, the continuous output voltages of ring oscillators consisting of FBFETs can be extracted using the LIM functions. These LIM functionalities are advantageous for implementing advanced PUF and TRNG applications. Therefore, in this paper, we propose the generation and storage of random voltages using ring oscillators. Random voltages can be derived from oscillatory cycles and can be preserved for several hundreds to thousands of seconds.

## 2. Simulation Methods

Electrical characteristics of FBFETs, inverters, and ring oscillators were simulated using a commercial two-dimensional device simulator (Synopsys Sentaurus (O_2018.06)) [22]. In these simulations, the physical models for FBFETs included Fermi–Dirac statistics and Slotboom bandgap narrowing models. For a detailed recombination analysis, doping-dependent Shockley–Read–Hall and Auger models were used. Moreover, we analyzed the silicon regions using models for both inversion and accumulation layer mobilities and high-field saturation mobility, with default parameters for all the models. The detailed simulation models and parameters are listed in Table S1, Supporting Information.

## 3. Results and Discussion

**Figure 1:**
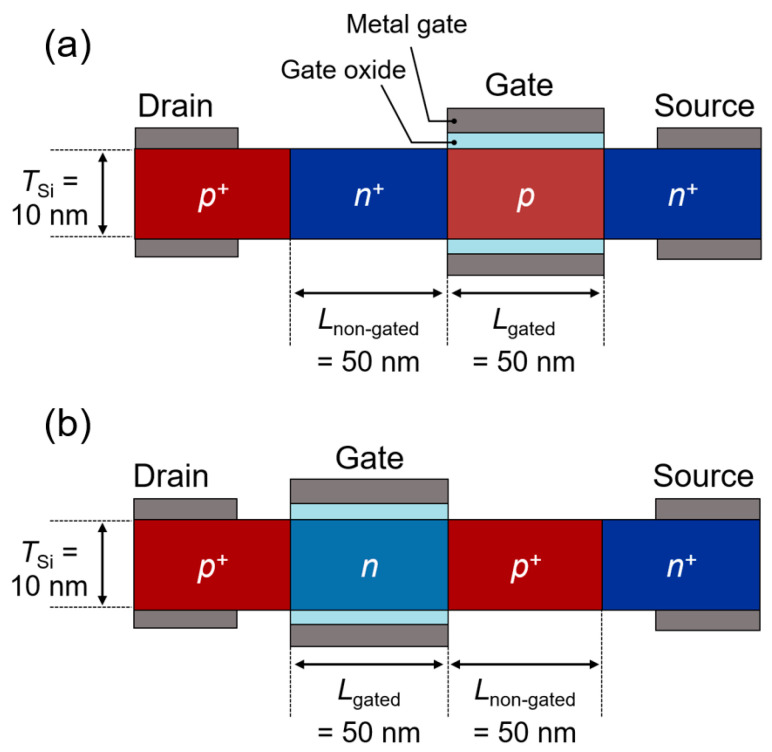
Cross-sectional schematics of (**a**) *n*-FBFET and (**b**) *p*-FBFET.

The cross-sectional views of an *n*-channel FBFET (*n*-FBFET) and a *p*-channel FBFET (*p*-FBFET) are shown in Figure 1a and Figure 1b, respectively. Both the FBFETs consisted of a *p*-type doped drain, an *n*-type doped source, and gated/nongated channel regions. Al_2_O_3_ gate oxide layers and metal gate electrodes (work function = 4.6 eV) were stacked on the top and bottom of the *p*-type doped channel for the *n*-FBFET and the *n*-type doped channel for the *p*-FBFET. These FBFETs had identical dimensional parameters and doping concentrations in each region. The gated channel length (*L*_gated_), nongated channel length (*L*_non-gated_), silicon channel thickness (*T*_Si_), and gate oxide thickness (*T*_ox_) were 50, 50, 10, and 3 nm, respectively. The doping concentration of the *p*-type doped drain, *n*-type doped source, and nongated regions (the *n*-type doped channel for the *n*-FBFET and the *p*-type doped channel for the *p*-FBFET) was 1 × 10^20^ cm^−3^. The doping concentration of the gated regions (the *p*-type doped channel for the *n*-FBFET and the *n*-type doped channel for the *p*-FBFET) was 8 × 10^19^ cm^−3^. The FBFETs were designed to accurately represent a 3D nanosheet gate-all-around structure. The silicon channel width (*W*_Si_) was set to be equal to the *T*_Si_ = 10 nm, and the gate electrodes at the top and bottom of the silicon channel were coupled together. Their *p*–*n*–*p*–*n* doping structures generate a positive feedback mechanism, which is a reciprocal interaction between the channel potential barriers and the charge carriers. The generation and elimination of a positive feedback loop in the channel region enable rapid switching. In the positive feedback loop, charge carriers accumulate in or are removed from the channel potential wells. The presence or absence of charge carriers in the channel regions results in bistable memory states, allowing FBFETs to operate as switchable-memory devices [23]. Furthermore, the FBFET structures are compatible with CMOS top-down fabrication techniques. The nano-scale silicon channels of the FBFETs can be achieved by stacking Si/SiGe multilayers [24]. The selective removal of the sacrificial SiGe layers enables the devices to be formed as a vertical nanosheet gate-all-around structure. Also, the *p*–*n*–*p*–*n* doping structures can be created using conventional photolithography and an ion implantation process, indicating that the fabrication of the silicon-based FBFETs is cost-efficient and straightforward.

**Figure 2:**
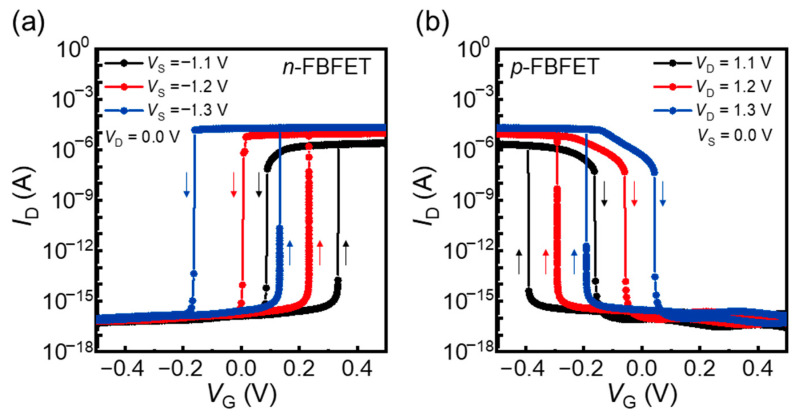
Transfer characteristics of (**a**) *n*-FBFET depending on *V*_S_ and (**b**) *p*-FBFET depending on *V*_D_.

Figure 2a,b shows the transfer characteristics of the *n*- and *p*-FBFETs, respectively. Negative source voltages (*V*_S_) and positive drain voltages (*V*_D_) were applied to the *n*- and *p*-FBFETs, respectively, based on the configurations of the inverters and ring oscillators. During the forward and reverse gate voltage (*V*_G_) sweeps, latch-up/latch-down phenomena were observed in both the *n*- and *p*-FBFETs, owing to the generation and elimination of the positive feedback loop in their channels. Both the *n*- and *p*-FBFETs exhibited an excellent switching performance, including ON/OFF current ratios (approximately 10^11^), low OFF currents (approximately 10^−16^ A), and extremely low subthreshold swings (<1 mV/dec). Furthermore, these FBFETs exhibited memory windows defined by the differences between the latch-up and latch-down voltages resulting from the accumulation of charge carriers in the channel potential wells during the positive feedback loop. The positions and widths of the memory windows can be adjusted using the supply voltages (*V*_D_ and *V*_S_) or *V*_G_. Considering the experimental fabrication environment, the lateral diffusion effect of the interfaces between the doping regions is investigated in Figure S1, Supporting Information.

On the other hand, the different electrical characteristics between the *n*- and *p*-FBFETs are caused by the different type of the minority charge carriers; the minority charge carriers of the *n*- and *p*-FBFETs are holes and electrons, respectively. These minority charge carriers are key to maintaining the positive feedback loop by continuously accumulating in the potential wells. Generally, electron recombination is much faster than hole recombination [25]. Consequently, the positive feedback loop in the *p*-FBFET exhibits a relatively lower strength compared to that of the *n*-FBFET. However, the rapid electron recombination in the *p*-FBFET can be mitigated by reducing the channel length (*L*_ch_) and increasing the *T*_Si_. A shorter *L*_ch_ enhances the charge carrier accumulation, and a thicker *T*_Si_ increases the amount of charge carriers to flow. Thus, the differences between the *n*- and *p*-FBFETs can be alleviated by adjusting the channel dimension parameters, *T*_Si_ and *L*_ch_.

**Figure 3:**
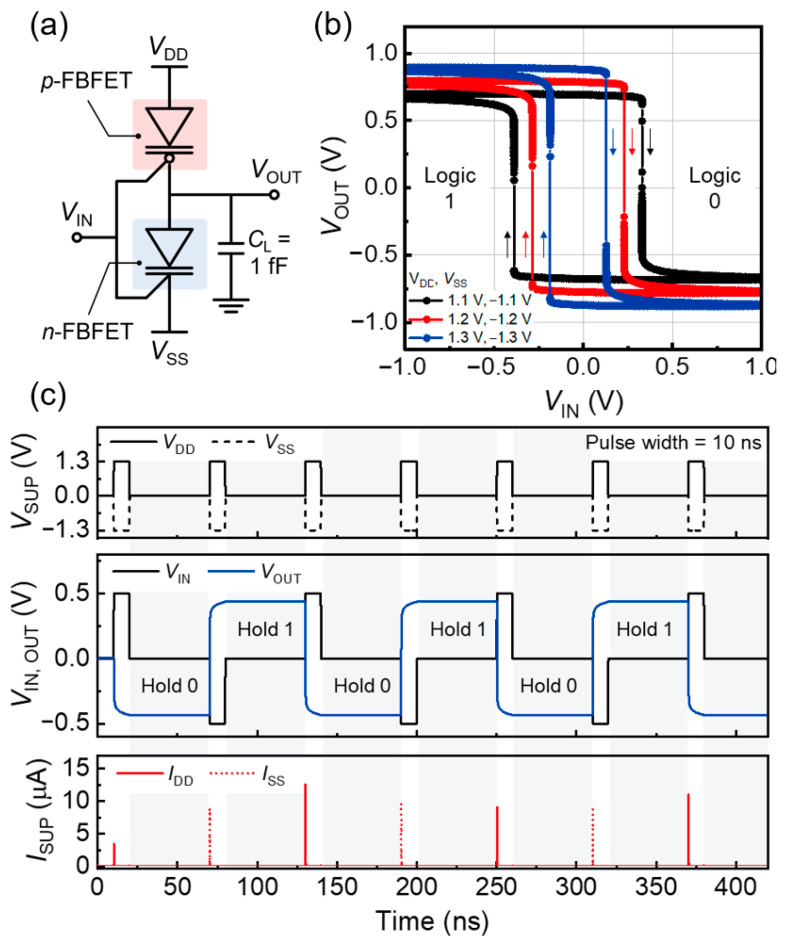
(**a**) Schematic of an inverter consisting of *n*- and *p*-FBFETs. (**b**) VTCs and (**c**) dynamic LIM operation of the inverter.

To evaluate the memory and logic capabilities, we configured the *n*- and *p*-FBFETs into a standard inverter circuit, as depicted in Figure 3a. A parasitic load capacitor (*C*_L_) of 1 fF was connected to the output node to reflect the output capacitances of the inverter and the interconnection line capacitances between the logic gates. Figure 3b shows the static voltage transfer characteristics (VTC) of the inverter. The inverter exhibited a notably high inverter gain with the maximum gain estimated as 3.56 × 10^5^ V/V at *V*_DD_ = 1.3 V and *V*_SS_ = −1.3 V. In forward and reverse input voltage (*V*_IN_) sweeps, clockwise voltage memory windows were observed owing to the inherent memory characteristics of FBFETs. The inverter can maintain the logic states within the *V*_IN_ range that corresponds to the memory windows; logic 0 and 1 are held during the forward and reverse *V*_IN_ sweeps, respectively. For the supply voltages, symmetrical positive *V*_DD_ and negative *V*_SS_ values were selected to ensure that the memory window included a *V*_IN_ of 0.0 V. The voltage condition of *V*_IN_ = 0.0 V was used for the memory operation of the inverter, enabling the minimization of energy consumption. As for the *V*_OUT_ values, the voltage values of logic 0 and 1 were negative and positive, respectively, because of the use of positive *V*_DD_ and negative *V*_SS_ values.

The inverter performs LIM operations using a memory window within its static VTC. Figure 3c shows the dynamic *V*_OUT_ responses under a sequence of voltage pulses of logic 0 and 1 with the supply voltages set at *V*_DD_ = 1.3 V and *V*_SS_ = −1.3 V. Following each logic pulse of 10 ns, the supply voltages (*V*_DD_ and *V*_SS_) and *V*_IN_ are reset to 0.0 V for the hold operations. During the hold operations, the inverter maintains its logic state (logic 0 or 1) because of the accumulation of charge carriers in the channels of the component devices. This accumulation causes the component devices to be charged; the *n*- and *p*-FBFETs become charged after the voltage pulses of logic 0 and 1, respectively. Thus, the inverter effectively preserves the logic state without the need for an external bias. Furthermore, FBFETs exhibit quasi-nonvolatile memory characteristics with a duration of hundreds of seconds, surpassing the performance of other charge-based memory transistors [26]. Their superior memory retention capabilities are detailed in our previous work [27,28].

**Figure 4:**
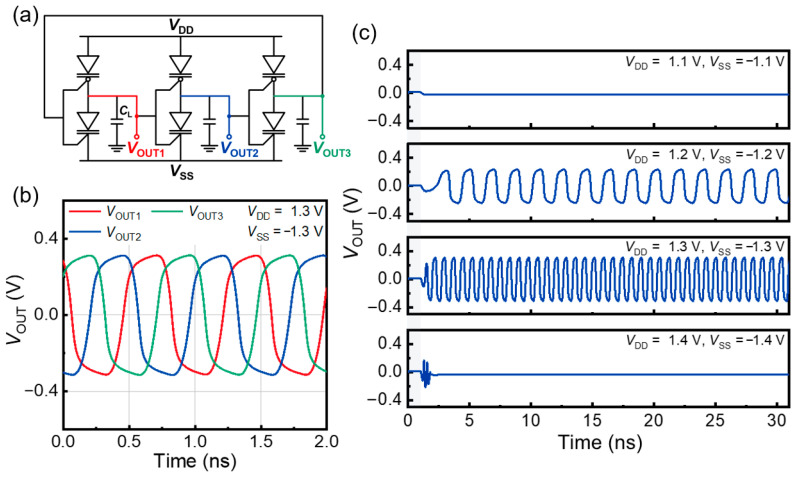
(**a**) Schematic of a three-stage ring oscillator consisting of *n*- and *p*-FBFETs. (**b**) Transient output characteristics of each stage at *V*_DD_ = 1.3 V and *V*_SS_ = −1.3 V and (**c**) under various supply voltages.

The capability of the FBFETs to maintain the *V*_OUT_ values offers diverse applications when integrated into logic cascading levels beyond a single-inverter circuit. In particular, in oscillatory operations where the output voltages vary continuously, the LIM functions of the FBFETs can be utilized to generate various *V*_OUT_ values. To explore the oscillatory behavior of the FBFETs, we configured a three-stage ring oscillator by connecting three inverters, as shown in Figure 4a. Each inverter was connected in sequence, and the output of the final inverter was fed back to the first input to achieve continuous oscillations. The parasitic *C*_L_s at the output nodes of each inverter were set to 1 fF. Figure 4b shows the transient output characteristics of the three-stage ring oscillator at *V*_DD_ = 1.3 V and *V*_SS_ = −1.3 V. Each stage of the output node voltages exhibited self-sustained oscillations that ranged from −0.4 V to 0.4 V with a phase shift of 2π/3 (120°) during oscillation, which is attributed to a π/3 phase shift from each inverter and a π phase shift from static inversion. The ring oscillator frequency (*f*_RO_) was obtained using the equation *f*_RO_ = (2 × *n* × *T*_d_)^–1^, where *n* is the number of stages, and *T*_d_ is the inverter stage delay. For this ring oscillator, *T*_d_ and *f*_RO_ are estimated to be 0.75 ns and 220 MHz, respectively.

The transient output characteristics of the ring oscillator at various supply voltages are shown in Figure 4c. When the absolute values of the supply voltages were set at 1.1 V, oscillation did not occur because the *V*_OUT_ levels of each inverter were insufficient to serve as *V*_IN_ for the subsequent inverter stages. By contrast, at the absolute supply voltage values of 1.2 V or 1.3 V, the ring oscillator began to oscillate, owing to the adequate *V*_OUT_ levels of each inverter. The frequency of these oscillations can be modulated by varying the supply voltage, allowing the system to function as a voltage-controlled oscillator [29]. However, when the absolute values of the supply voltages exceeded 1.4 V, both the *n*- and *p*-FBFETs were turned ON simultaneously, thereby interrupting the oscillation. The excessive accumulation of charge carriers in the channel regions of the FBFETs at high supply voltages interrupts the oscillation. 

**Figure 5:**
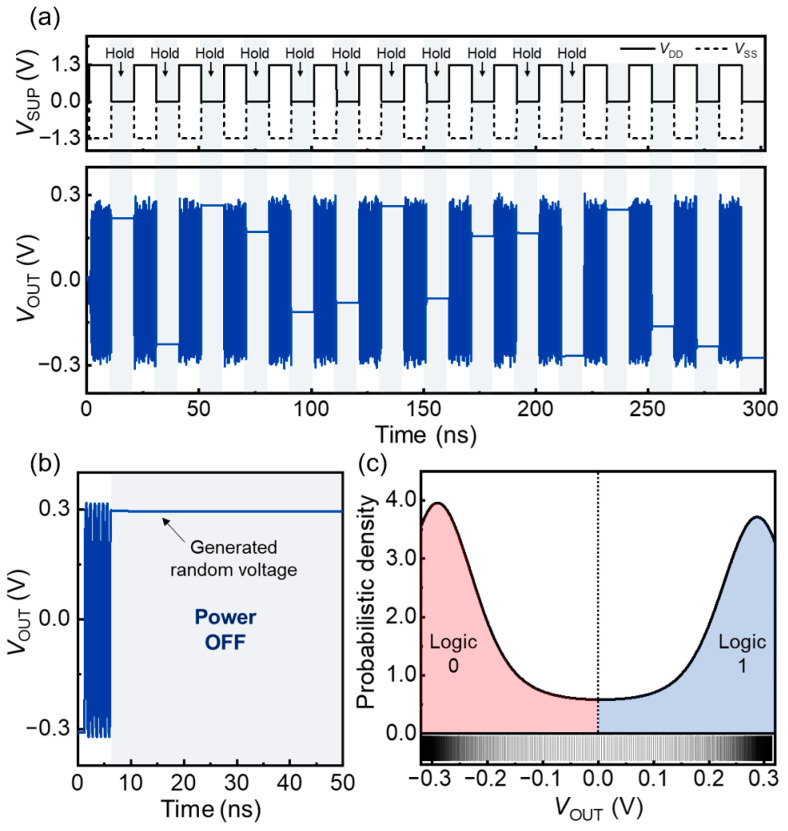
(**a**) Overall and (**b**) detailed memory operations of the ring oscillator under repetitive power ON/OFF cycles. (**c**) Probability density of *V*_OUT_ values extracted from the oscillatory cycles.

Analog voltages were randomly generated by the ring oscillator. Figure 5a,b shows the *V*_OUT_ values of the ring oscillator as a function of time under repetitive power ON (*V*_DD_ = 1.3 V and *V*_SS_ = −1.3 V) and power OFF (*V*_DD_ = *V*_SS_ = 0.0 V) cycles; in this figure, for the hold operation, the power is OFF. The *V*_OUT_ values depend on the number of charge carriers accumulated in the channels of the *n*- and *p*-FBFETs, and these values are preserved by the component inverters when the power is switched OFF. When the power was restored, the ring oscillator immediately returned to its oscillatory state without warm-up time. Consequently, the ring oscillator generated and stored random analog voltage values. 

Like other hardware-based TRNG and PUF circuits, the randomness of the FBFET-based ring oscillator comes from the variation in the fabrication and internal latency. Due to the nature of the processes, the speed and amount of the charge carrier accumulation in the FBFET channels differ slightly for every operation, which leads to latency variations. Also, the irregular timing of the power ON/OFF cycles is a random factor for our ring oscillator. Figure 5c illustrates the probability density distribution of the *V*_OUT_ values extracted from the oscillatory cycles of the ring oscillator. In the distribution, the probability density is congregated at the crest and trough of the oscillation (−0.3 V and 0.3 V) owing to the sine waveform of the oscillation. To use the *V*_OUT_ of the ring oscillator as a random variable, the probabilities of the variables should be uniformly distributed and possess a wide bandwidth [15,30]. Therefore, we divided the probability distribution of the *V*_OUT_ values into two distinct domains: negative and positive *V*_OUT_ domains, representing logic 0 and 1, respectively. Using this approach, the probabilities of logic 0 and 1 were estimated to be 51.6% and 48.4%, respectively, demonstrating a highly uniform distribution between the two domains. This configuration enabled the ring oscillator to function effectively as a TRNG.

**Figure 6:**
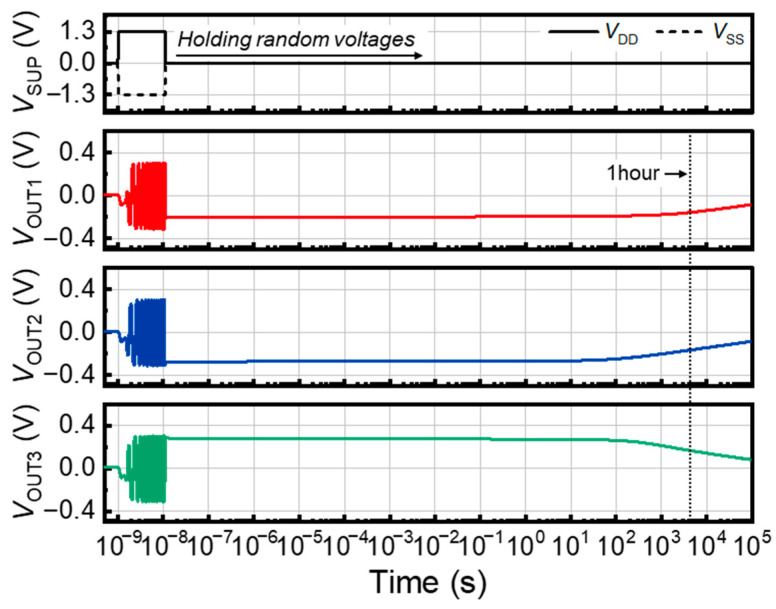
*V*_OUT_ retention properties of the ring oscillator.

The retention times of the random voltages generated by the oscillations were analyzed to validate the memory stability of the ring oscillator. Figure 6 shows the *V*_OUT_ retention times of each inverter stage in the three-stage ring oscillator. To generate random voltage signals, supply voltage pulses with durations of 10 ns were applied to the ring oscillator. Following the supply voltage pulses, all the external biases were reset to 0.0 V for 10^5^ s to verify the *V*_OUT_ retention times. During the hold operation, the *V*_OUT_ values of the ring oscillator decreased toward 0.0 V, owing to the loss of accumulated charge carriers of the component devices. The ring oscillator effectively maintained the *V*_OUT_ value for an initial holding time of 200 s, and the average degradation of the *V*_OUT_ value was only 10.6%. The retention time was defined as the time at which the initial *V*_OUT_ value decreased to 63% of its original value following the standard time constant principle [31]. The average *V*_OUT_ retention time of the ring oscillator was estimated to be approximately 5500 s, demonstrating robust memory stability. On the other hand, the *V*_OUT_ retention time of the ring oscillator is affected by the *T*_Si_ (Figure S2, Supporting Information). As the *T*_Si_ thickens, the average *V*_OUT_ retention time decreases. This decrease is due to the bulk recombination, which intensifies with an increase in the channel depth [32]. As a result, thinner *T*_Si_ is required to provide sufficient *V*_OUT_ retention time. Nevertheless, the ring oscillator generates analog random voltage signals and stores their values with a significant retention time. These operations are beneficial for security applications, offering a robust method for generating and storing PUF keys in several ring oscillators [33,34].

## 4. Conclusions

This study demonstrated the generation and storage of random voltage values with a ring oscillator consisting of *n*- and *p*-FBFETs using computer-aided design simulations. The ring oscillator exhibited dual capabilities of generating and storing random voltages during power ON/OFF cycles by utilizing the LIM functions of the component devices. The probability distribution of the *V*_OUT_ values of the ring oscillator can be divided into two distinct domains: logic 0 and 1. The probabilities were close to 50%. Moreover, the ring oscillator demonstrated the self-storage capability of random variables, with a retention time of approximately 5500 s.

## Data Availability

Data are contained within the article and Supplementary Materials.

## Supplementary Materials

The following supporting information can be downloaded at: https://www.mdpi.com/article/10.3390/nano14070562/s1, Table S1. Detailed simulation models and parameters; Table S2. Randomness test results of the FBFET-based ring oscillator under NIST SP800-22 test suite; Figure S1. Schematic diagrams and transfer characteristics of (a) n-FBFETs and (b) p-FBFETs with various gaussian doping profiles; Figure S2. Positive and negative VOUT retention properties of ring oscillators with various TSi.
