# Supplementary material for: Generation and Storage of Random Voltage Values via Ring Oscillators Comprising Feedback Field-Effect Transistors
Source: Nanomaterials (Basel). 2024 Mar 23;14(7):562. doi: 10.3390/nano14070562 (PMC11013403; doi:10.3390/nano14070562)
Supplement: Supplementary file 1 [file nanomaterials-14-00562-s001.zip › nanomaterials-2901060-supplementary.pdf]

Supporting Information

Generation and Storage of Random Voltage Values via Ring Oscillators Comprising Feedback Field-Effect Transistors

Jaemin Son, Juhee Jeon, Kyoungah Cho * and Sangsig Kim *

Department of Electrical Engineering, Korea University, 145 Anam-ro, Seongbuk-gu, Seoul 02841, Republic of Korea; jaeminson@korea.ac.kr (J.S.); isdf35@korea.ac.kr (J.J.)

* Correspondence: chochem@korea.ac.kr (K.C.); sangsig@korea.ac.kr (S.K.); Tel.: +82-2-3290-3245 (S.K.); Fax: +82-2-3290-3894 (S.K.)

Table S1. Detailed simulation models and parameters.

Type	Model	Equation	Parameter
Carrier statistics	Fermi-dirac statistics	$n = N_C F_{1/2}\left(\frac{E_{F,n} - E_C}{kT}\right),$<br>$p = N_V F_{1/2}\left(\frac{E_V - E_{F,p}}{kT}\right)$	-
Effective intrinsic density	Bandgap narrowing (Slotboom)	$\Delta E_g^0 = E_{ref}\left[\ln\left(\frac{N_{tot}}{N_{ref}}\right) + \sqrt{\left(ln\left(\frac{N_{tot}}{N_{ref}}\right)\right)^2 + 0.5}\right]$	E_{ref} = 6.92×10^{-3},<br>N_{ref} = 1.3×10^{17}
Carrier mobility	Inversion and accumulation layer mobility	Electron mobility :<br>$c = n, c_{other} = p, N_{inv} = N_{A,0}, N_{acc} = N_{D,0},$<br>$P = P_e, m^* = m_e^*, m_{other}^* = m_h^*$<br>Hole mobility :<br>$c = p, c_{other} = n, N_{inv} = N_{D,0}, N_{acc} = N_{A,0},$<br>$P = P_h, m^* = m_h^*, m_{other}^* = m_e^*$	$N_{D,ref}$ = 4×10^{20} cm^{-3},<br>$N_{A,ref}$ = 7.2×10^{20} cm^{-3},<br>c_D = 0.21,<br>c_A = 0.5,<br>m_e^*/m_0 = 1.0,<br>m_h^*/m_0 = 1.0,
	High field saturation (basic)	$\mu = \mu_{low}\left(\frac{300\ K}{T_c}\right)$	-
Recombi-nation and generation	Shockley-read-hall (doping dependency)	$\tau_{dop}(N_{A,0} + N_{D,0}) = \tau_{min} + \frac{\tau_{max} - \tau_{min}}{1 + \left(\frac{N_{A,0} + N_{D,0}}{N_{ref}}\right)^{\gamma}}$	τ_{min}= 0(e), 0(h)<br>τ_{max}= 1×10^{-5} s(e), 3×10^{-6} s(h)<br>N_{ref} = 1×10^{16} cm^{-3}(e),<br>1×10^{16} cm^{-3}(h)<br>γ= 1
	Auger	$R_{net}^A = (C_n n + C_p p)(np - n_{i,eff}^2),$<br>$C_n(T) = \left(A_{A,n} + B_{A,n}\left(\frac{T}{T_0}\right) + C_{A,n}\left(\frac{T}{T_0}\right)^2\right)\left[1 + H_n exp\left(-\frac{n}{N_{0,n}}\right)\right],$<br>$C_n(T) = \left(A_{A,p} + B_{A,p}\left(\frac{T}{T_0}\right) + C_{A,p}\left(\frac{T}{T_0}\right)^2\right)\left[1 + H_p exp\left(-\frac{p}{N_{0,p}}\right)\right]$	$A_{A,n}$ = 6.7×10^{-32} cm^{-3}s^{-1},<br>$A_{A,h}$ = 7.2×10^{-32} cm^{-3}s^{-1},<br>$C_{A,n}$ = –2.2×10^{-32} cm^{-3}s^{-1},<br>$C_{A,h}$ = –2.2×10^{-32} cm^{-3}s^{-1},<br>H_e = 3.46667,<br>H_h = 8.25688,<br>$N_{0,e}$ = 1×10^{18} cm^{-3},<br>$N_{0,h}$ = 1×10^{18} cm^{-3}

Table S2. Randomness test results of the FBFET-based ring oscillator under NIST SP800-22 test suite.

Test type	P-value	Result
Frequency (Monobit)	0.7962534147376393	Random
Frequency within a Block	0.7962534147376393	Random
Runs	0.18883007821588327	Random
Non-overlapping Template Matching	0.9992522603315008	Random
Serial	0.4989610874592239	Random
Approximate Entropy	1.0	Random
Cumulative Sums (Forward)	1.0388250053759687	Random
Cumulative Sums (Backward)	0.8470584065743881	Random
Random Excursions	0.9821754508742055	Random
Random Excursions Variant	0.7150006546880893	Random

S1. Electrical characteristics of FBFETs regarding lateral diffusion of dopants

Figure S1(a) and S1(b) illustrate schematic diagrams and transfer characteristics for the *n*- and *p*-FBFETs with varying gaussian doping profiles, respectively. These gaussian doping profiles simulate the lateral diffusion of dopants. As the lateral diffusion intensifies, the memory window widths for both the *n*- and *p*-FBFETs increase due to the reduced length of channel doping regions. Nevertheless, the FBFETs consistently demonstrated robust switching and memory capabilities even when the lateral diffusion is intensified.

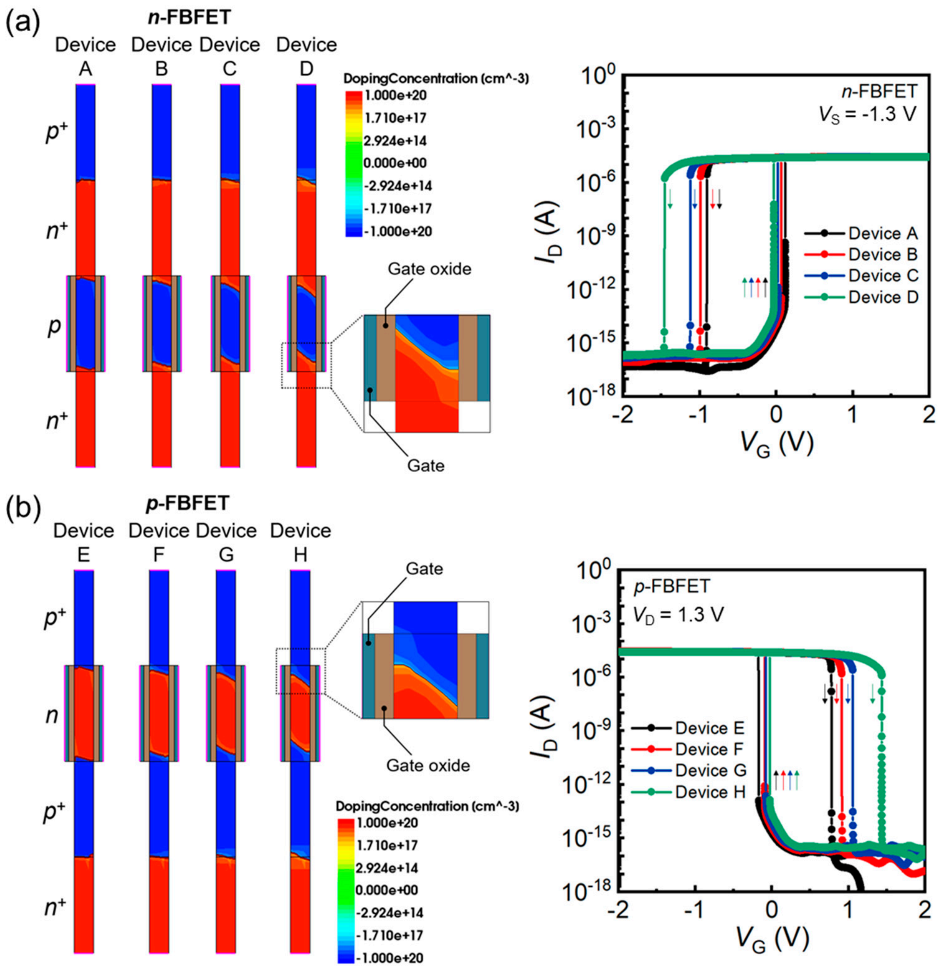

Figure S1. Schematic diagrams and transfer characteristics of (**a**) *n*-FBFETs and (**b**) *p*-FBFETs with various gaussian doping profiles.

S2. Retention characteristics of ring oscillator depending on FBFET channel thickness

Figure S2 shows both the positive and negative V_{OUT} retention properties of the ring oscillators with varying FBFET channel thickness (T_{Si}). As the T_{Si} increases, the average V_{OUT} retention time of the ring oscillator decreases; the average retention times are estimated to be 5500 s for T_{Si} = 10 nm, 1363 s for T_{Si} = 30 nm, and 638 s for T_{Si} = 50 nm.

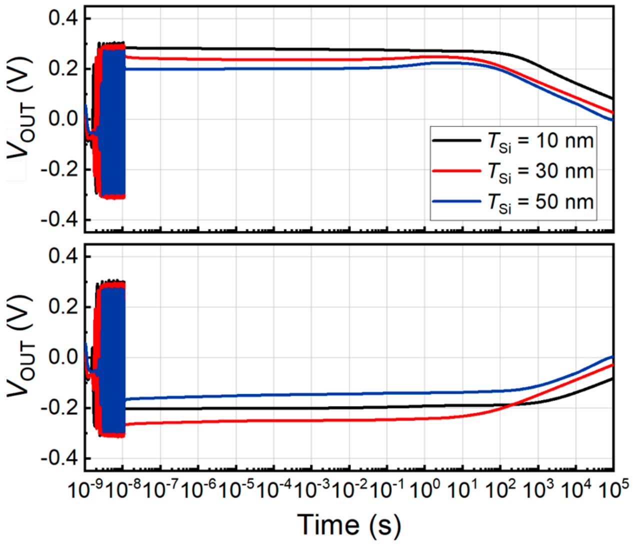

Figure S2. Positive and negative V_{OUT} retention properties of ring oscillators with various T_{Si}
